# Routine transthoracic echocardiography in ischaemic stroke or transient ischaemic attack of undetermined cause: a prospective multicentre study

**DOI:** 10.1007/s12471-023-01819-7

**Published:** 2023-10-23

**Authors:** Gerlinde van der Maten, Matthijs F. L. Meijs, Jorik R. Timmer, Paul J. A. M. Brouwers, Clemens von Birgelen, Jonathan M. Coutinho, Berto J. Bouma, Henk Kerkhoff, Anne Mijn Helming, Julia H. van Tuijl, Nicolet A. van der Meer, Ritu Saxena, Corné Ebink, Job van der Palen, Heleen M. den Hertog

**Affiliations:** 1 Department of Neurology, Medisch Spectrum Twente, Enschede, The Netherlands; 2 Department of Health Technology and Services Research, Faculty of Behavioural, Management and Social Sciences, University of Twente, Enschede, The Netherlands; 3 Department of Cardiology, Medisch Spectrum Twente, Enschede, The Netherlands; 4 Department of Cardiology, Isala Hospital, Zwolle, The Netherlands; 5 Department of Neurology, Amsterdam University Medical Centres, location AMC, Amsterdam, The Netherlands; 6 Department of Cardiology, Amsterdam University Medical Centres, location AMC, Amsterdam, The Netherlands; 7 Department of Neurology, Albert Schweitzer Hospital, Dordrecht, The Netherlands; 8 Department of Cardiology, Albert Schweitzer Hospital, Dordrecht, The Netherlands; 9 Department of Neurology, Elisabeth-TweeSteden Hospital, Tilburg, The Netherlands; 10 Department of Cardiology, Elisabeth-TweeSteden Hospital, Tilburg, The Netherlands; 11 Department of Neurology, Maasstad Hospital, Rotterdam, The Netherlands; 12 Department of Cardiology, Maasstad Hospital, Rotterdam, The Netherlands; 13 Section Cognition, Data and Education, Faculty of Behavioural, Management and Social Sciences, University of Twente, Enschede, The Netherlands; 14 Medical School Twente, Medisch Spectrum Twente, Enschede, The Netherlands; 15 Department of Neurology, Isala Hospital, Zwolle, The Netherlands

**Keywords:** Brain ischaemia, Echocardiography, Embolism, Transient ischaemic attack

## Abstract

***Background*:**

Guidelines recommend routine transthoracic echocardiography (TTE) after ischaemic stroke or transient ischaemic attack of undetermined cause; yet, only limited scientific evidence exists. Therefore, we aimed to determine in these patients the prevalence of TTE-detected major cardiac sources of embolism (CSE), which are abnormalities leading to therapeutic changes.

***Methods*:**

Six Dutch hospitals conducted a prospective observational study that enrolled patients with ischaemic stroke or transient ischaemic attack of undetermined cause. Patients underwent TTE after comprehensive diagnostic evaluation on stroke units, including blood chemistry, 12-lead electrocardiogram (ECG), ≥ 24 h continuous ECG monitoring, brain imaging and cervical artery imaging. Primary outcome measure was the proportion of patients with TTE-detected major CSE.

***Results*:**

From March 2018 to October 2020, 1084 patients, aged 66.6 ± 12.5 years, were enrolled; 456 (42.1%) patients were female and 869 (80.2%) had ischaemic stroke. TTE detected major CSE in only 11 (1.0%) patients. Ten (90.9%) of these patients also had major ECG abnormalities (previous infarction, major repolarisation abnormalities, or previously unknown left bundle branch block) that would have warranted TTE assessment regardless of stroke evaluation. Such ECG abnormalities were present in 11.1% of the total study population. A single patient (0.1%) showed a major CSE despite having no ECG abnormality.

***Conclusions*:**

This multicentre cross-sectional study in patients who—after workup on contemporary stroke units—were diagnosed with ischaemic stroke or transient ischaemic attack of undetermined cause found TTE-detected major CSE in only 1% of all patients. Most of these patients also had major ECG abnormalities. These findings question the value of routine TTE assessment in this clinical setting.

**Supplementary Information:**

The online version of this article (10.1007/s12471-023-01819-7) contains supplementary material, which is available to authorized users.

## What’s new?

- Scientific evidence on the role of routine transthoracic echocardiography (TTE) to detect a major cardiac source of embolism (CSE) in patients with ischaemic stroke of undetermined cause is limited.
- This large-scale multicentre study showed that routine TTE infrequently detects a major CSE in patients with ischaemic stroke or transient ischaemic attack of undetermined cause.
- The vast majority of patients with a major CSE also had major ECG abnormalities.
- Our findings suggest that the current referral strategy for echocardiography might be replaced by selective echocardiography in order to reduce both pressure on the healthcare system and unnecessary costs.

## Introduction

After ischaemic stroke, diagnostic evaluation aims to determine the probable cause to guide treatment choices that reduce the risk of recurrent stroke [1]. Despite diagnostic workup, the cause of ischaemic stroke remains undetermined in about 25% of patients [1]. These patients are often classified according to the Trial of Org 10,172 in Acute Stroke Treatment (TOAST) criteria [2], whereas the term embolic stroke of undetermined source (ESUS) is reserved for a subset of patients with presumed embolic stroke who have had sufficient evaluation to rule out a major cardiac source of embolism (CSE), relevant atherosclerosis, or lacunar infarct [3].

When no cause of stroke is identified, echocardiographic assessment is used to search for a major or minor CSE [4]. A major CSE does have a clear pathophysiological relation to ischaemic stroke, and the appropriate change of therapy reduces the risk of recurrent stroke. In contrast, a minor CSE might be potentially related to ischaemic stroke but has no therapeutic consequences [4].

Routine use of echocardiography in patients with ischaemic stroke may seem straightforward. Nevertheless, guidelines offer contradicting recommendations. The European Stroke Organisation implicitly suggests that transthoracic echocardiography (TTE) be performed in all ischaemic stroke patients [5], whereas the European Association of Cardiovascular Imaging (EACVI) recommends performing contrast TTE in patients without carotid stenosis of > 70% [4] and the Dutch national stroke guideline advises TTE if no cause of stroke has been identified [6]. Nonetheless, evidence to support these recommendations is limited. A recently published systematic review and meta-analysis showed that after ischaemic stroke of undetermined cause, major CSE are infrequently detected with TTE [7]. In addition, although TTE might be relatively inexpensive, its current availability in the Netherlands is limited due to a shortage of echocardiographers. Consequently, performing TTE on a routine basis might be at the expense of patients with more urgent indications. We therefore aimed to determine the prevalence of major CSE using routine TTE in patients with ischaemic stroke or transient ischaemic attack (TIA) of undetermined cause.

## Methods

**Fig. 1:**
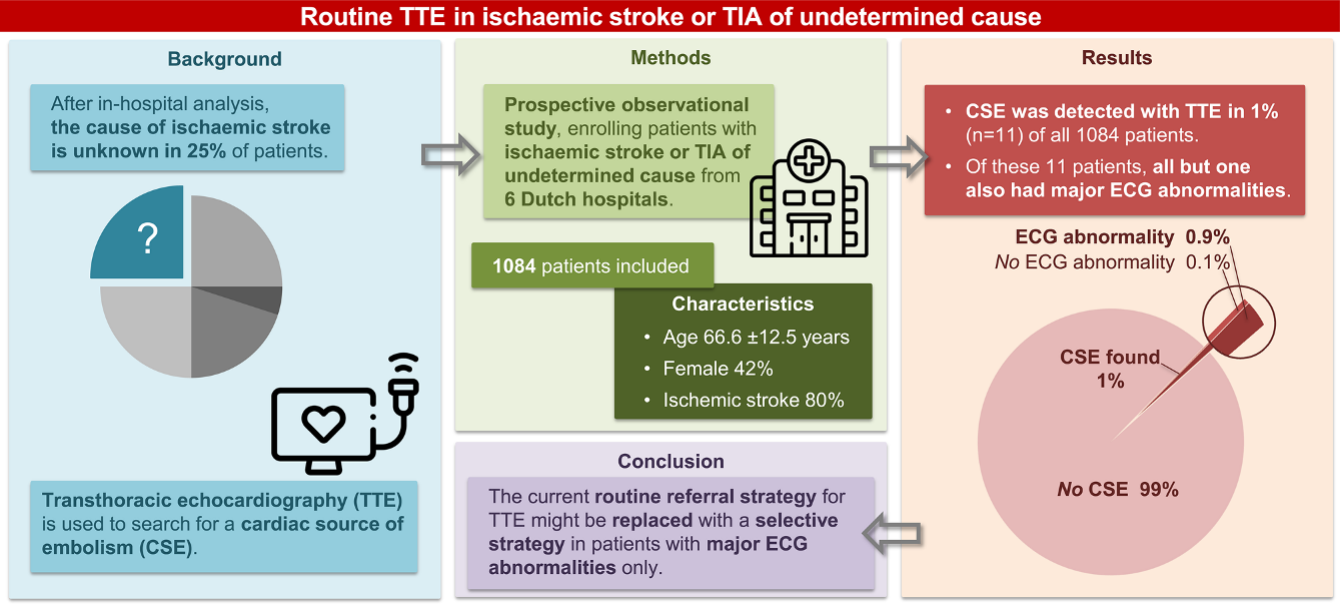
Infographic presenting an overview of the study design and main results. *TIA* transient ischaemic attack

An overview of the study design and main results is presented in Fig. 1.

### Study design and participants

Between March 2018 and October 2020, we performed a cross-sectional study that consecutively enrolled patients from inpatient and outpatient clinics of six Dutch hospitals (Table S1, Electronic Supplementary Material). Patients were eligible if they primarily presented to one of the participating hospitals and had ischaemic stroke or TIA of undetermined cause after standard diagnostic evaluation. This evaluation consisted of a medical history, physical examination, 12-lead electrocardiogram (ECG), continuous ECG monitoring for ≥ 24 h, routine blood chemistry, brain imaging with computed tomography (CT) and/or magnetic resonance imaging (MRI), and cervical artery imaging using Doppler ultrasonography, CT angiography and/or MR angiography. Patients were eligible for inclusion regardless of potential additional outpatient cardiac rhythm monitoring results performed in parallel with TTE. Ischaemic stroke or TIA of undetermined cause was defined according to TOAST criteria [2]. Signs of previous myocardial infarction (pathological Q waves, abnormal R‑wave progression), major repolarisation abnormalities (suggestive of coronary artery disease or cardiomyopathy) and previously unknown left bundle branch block were considered as major ECG abnormalities.

This study was reviewed by the Medical Ethics Committee Twente in Enschede, the Netherlands (K18-08); it was not considered to be subject to the Medical Research Involving Human Subjects Act. According to the Dutch General Data Protection Regulation, informed consent was obtained. This study was prospectively registered with the Netherlands Trial Register, registration number NTR6993.

### TTE methods

All patients had TTE assessment performed by a certified sonographer. Patients were imaged in the left lateral decubitus position. Long-axis, short-axis, two-chamber and four-chamber images were obtained in the parasternal and apical views, and stored together with colour Doppler and pulsed and continuous wave Doppler data. If appropriate, intravenous agitated saline contrast was injected for the detection of a patent foramen ovale (PFO) [8]. Cardiologists analysed the images for left ventricular (LV) function, LV regional wall motion abnormalities, valvular disease, atrial volume and presence of thrombi or tumours. Echocardiographic findings were classified according to EACVI recommendations [4, 9]. Based on current evidence of treatment implications, CSE were divided into major and minor CSE (Table S2, Electronic Supplementary Material) [10–14].

PFO was regarded as a separate category. A possible relation with ischaemic stroke and consequent benefit from PFO closure is anticipated only in a highly selected group of young patients with certain stroke and PFO characteristics [15]. Following the recommendations of the Dutch guideline on PFO closure, patients < 60 years old with a risk of paradoxical embolism (RoPE) score of ≥ 6 were considered possible candidates for PFO closure [16]. In these patients, TTE or transoesophageal echocardiography (TOE) with agitated saline contrast was advised. Whether contrast was applied was decided by the treating cardiologist.

For assessment of inter-observer agreement, 50 TTEs were reviewed by a second and third cardiologist regarding the presence of major or minor CSE and, if examined, PFO. All cases showing a major CSE were selected; the remaining cases were randomly selected.

### Primary and secondary outcome measures

The primary outcome measure was the proportion of patients with a major CSE on TTE. Secondary outcome measures were the proportion of patients with a minor CSE on TTE and the prevalence of PFO in patients in whom its presence was considered relevant.

### Sample size calculation

At the start of the study, a pre-defined secondary outcome measure was the development of a prediction model. Based on an expected major CSE prevalence of 10%, a multivariable logistic regression model with 8 degrees of freedom required 1300 patients. However, after a pre-specified interim analysis, the prevalence of major CSE was low: 0.5% in 373 patients. Therefore, development of a prediction model was considered not feasible. After discussion with our independent, national funding organisation (The Netherlands Organisation for Health Research and Development, ZonMw), the sample size was reduced to 1000 patients. With an expected proportion of major CSE of 0.5–1.0%, this sample size would produce a two-sided 95% confidence interval (CI) with a width equal to 1.0–1.3%.

### Statistical analysis

All outcome measures were calculated with a 95% CI. Data were presented as numbers and frequencies, mean and standard deviation (SD) for continuous variables, and median and interquartile range (IQR) for categorical variables. We performed additional exploratory analyses to search for statistically significant differences between patients with and without a major CSE using an independent *t*-test, Mann-Whitney U test, chi-square test, or Fisher exact test, as appropriate. A significance level of 0.05 was used. Missing data were handled with list-wise deletion. All analyses were performed with IBM SPSS Statistics, version 24 (IBM Corp., Armonk, NY, USA).

## Results

### Study population

**Fig. 2:**
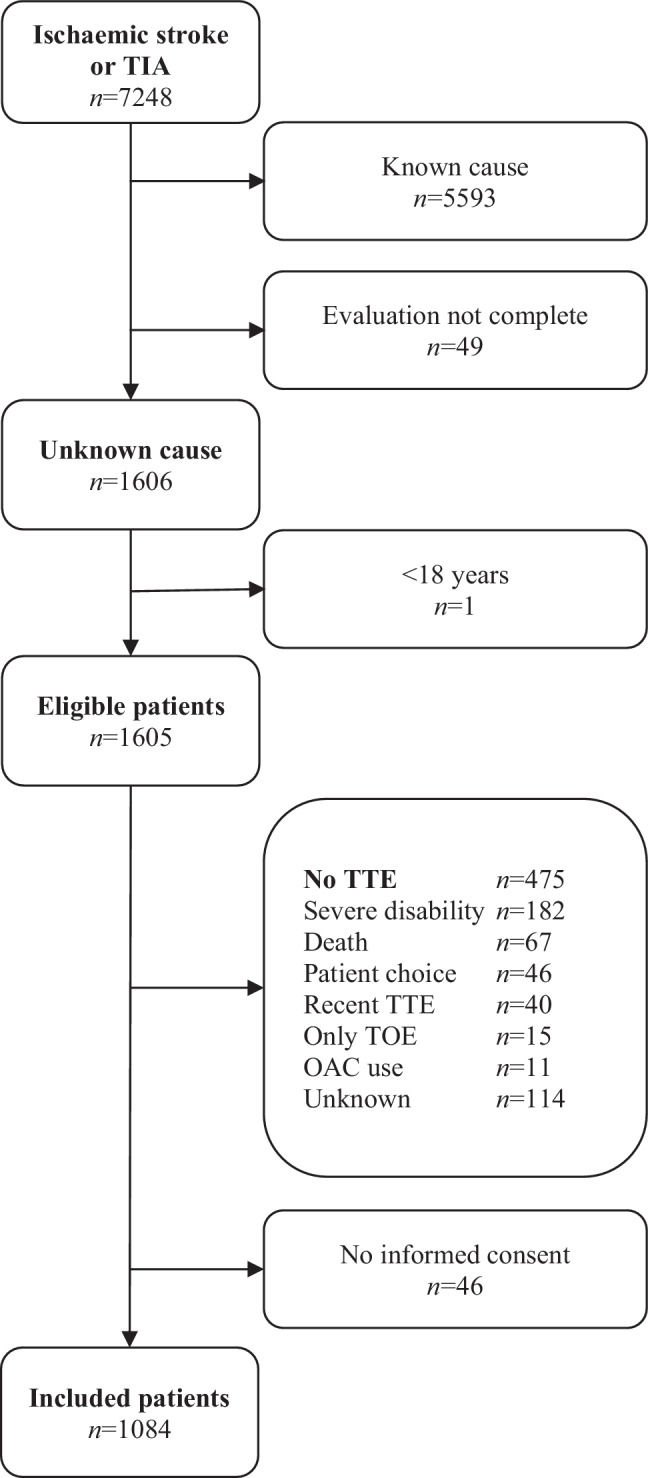
Patient inclusion. *TIA* transient ischaemic attack, *TTE* transthoracic echocardiography, *TOE* transoesophageal echocardiography, *OAC* oral anticoagulants

The enrolment process is summarised in Fig. 2. Of the 7248 patients with ischaemic stroke or TIA who presented to one of the participating hospitals, 1606 (22.1%) had ischaemic stroke or TIA of undetermined cause. A total of 1084 (67.5%) of these patients who underwent TTE were included. Mean age was 66.6 (SD 12.5) years, 456 (42.1%) patients were female and 869 (80.2%) had ischaemic stroke. The median interval between hospital admission and TTE was 30 days (IQR 4–47). Further patient characteristics are presented in Tab. 1.

**Table 1:** Characteristics of the study population

	Total (*n* = 1084)	CSE (*n* = 11)	No CSE (*n* = 1073)	*p*
Female	456 (42.1%)	4 (36.4%)	452 (42.1%)	0.476
Age (years)	66.6 ± 12.5	68.6 ± 10.2	66.6 ± 12.5	0.589
Young stroke (< 45 years)	59 (5.4%)	0 (0%)	59 (5.5%)	0.539
*Cardiovascular risk factors*				
Diabetes	180 (16.6%)	3 (27.3%)	177 (16.5%)	0.271
Dyslipidaemia	367 (33.9%)	8 (72.7%)	359 (33.5%)	* 0.009*
Hypertension	500 (46.1%)	6 (54.5%)	494 (46.0%)	0.573
Positive family history	186 (17.2%)	2 (18.2%)	184 (17.1%)	0.588
Smoking	217 (20.0%)	2 (18.2%)	215 (20.0%)	0.617
*History of*:				
Ischaemic stroke	187 (17.3%)	2 (18.2%)	185 (17.3%)	0.591
Cardiac disease	213 (19.7%)	8 (72.7%)	205 (19.1%)	*<* *0.001*
*Type of event*				
TIA	215 (19.8%)	1 (9.1%)	214 (19.9%)	0.326
Ischaemic stroke	869 (80.2%)	10 (90.9%)	859 (80.1%)	
*Acute treatment*	318 (29.3%)	7 (63.6%)	311 (29.0%)	* 0.019*
rtPA	288 (26.6%)	6 (54.5%)	282 (26.3%)	* 0.045*
Endovascular	92 (8.5%)	4 (36.4%)	88 (8.2%)	* 0.010*
Baseline NIHSS^a^	2 (1–5)	4.5 (1–19.3)	2 (1–5)	0.142
*Localisation*^b^				
Cortical	608 (56.1%)	9 (81.8%)	599 (55.8%)	
Subcortical	190 (17.5%)	1 (9.1%)	189 (17.6%)	
Cerebellar/brain stem	199 (18.4%)	2 (18.2%)	197 (18.4%)	
Multiple locations	45 (4.2%)	0 (0%)	45 (4.2%)	
Uncertain	42 (3.9%)	0 (0%)	42 (3.9%)	
*Brain imaging*				
Acute ischaemia on CT	230/1064 (21.6%)	2/11 (18.2%)	228/1053 (21.7%)	0.564
Hyperdense vessel sign	55/1064 (5.2%)	2/11 (18.2%)	53/1053 (5.0%)	0.107
Acute intracranial occlusion on CTA	142/534 (26.6%)	7/7 (100%)	135/527 (25.6%)	*<* *0.001*
Acute ischaemia on MRI	268/334 (80.2%)	1/1 (100%)	267/333 (80.2%)	0.802
*Vascular imaging*				
Doppler ultrasonography only	550 (50.7%)	4 (36.4%)	546 (50.9%)	0.257
CTA	534 (49.3%)	7 (63.6%)	527 (49.1%)	
Interval between hospital admission and TTE (days)	30 (4–47)	19 (1–41)	30 (4–47)	0.476
Major ECG abnormalities	119 (11.0%)	10 (90.9%)	109 (10.3%)	*<* *0.001*

Data are presented as *n* (%) except for age [mean (± SD)], NIHSS [median (Inter Quartile Range)] and interval between hospital admission and TTE [median (Inter Quartile Range)].

*CSE* cardiac source of embolism, *TIA* transient ischaemic attack, *rtPA* recombinant tissue plasminogen activator, *NIHSS* National Institute of Health Stroke Scale, *CT* computed tomography, *CTA* CT angiography, *MRI* magnetic resonance imaging, *TTE* transthoracic echocardiography, *ECG* electrocardiogram

^a^Patients with TIA excluded

^b^Localisation was determined by the results of both clinical examination and brain imaging

### Inter-observer agreement

After reviewing 50 TTEs, no clinically relevant conflicts arose between the judgements of the three reviewers. Inter-rater reliability values can be found in Table S3 (Electronic Supplementary Material).

### Summary of TTE results

TTE showed a major CSE in 11 patients (1.0%, 95% CI 0.5–1.7%) and a minor CSE in 29 patients (2.7%, 95% CI 1.8–3.8%). A possible or definite PFO was found in 27 of 117 patients (23.1%, 95% CI 16.2–31.3%) that underwent agitated saline contrast. Details of the most relevant TTE results are presented in Tab. 2. Basic TTE characteristics can be found in Table S4 (Electronic Supplementary Material) and the number of additional cardiac imaging studies is presented in Table S5 (Electronic Supplementary Material).

**Table 2:** Summary of transthoracic echocardiography results

*Major cardiac sources of embolism*	11 (1.0%)
Endocarditis	0
Intracardiac tumour	1 (0.1%)
LA/LAA thrombus	0
LV thrombus	3 (0.3%)
LV aneurysm	5 (0.5%)
LV thrombus and LV aneurysm	2 (0.2%)
Mitral valve stenosis	0
*Minor cardiac sources of embolism*	29 (2.7%)
Complex aortic arch atheromatous plaque	1 (0.1%)
LV dysfunction with LVEF < 35%	12 (1.1%)
Moderate or severe aortic valve stenosis	15 (1.4%)
LV dysfunction with LVEF < 35% and aortic valve stenosis	1 (0.1%)
*Right-to-left shunt*	
Patients < 60 years and RoPE ≥ 6	180/1084
Contrast echocardiography	117 (65.0%)
Definite PFO	26 (22.2%)
PFO closure	18 (15.4%)
Initiation of OAC	2 (1.7%)
No treatment change	6 (5.1%)
Possible PFO	1 (0.9%)

Data are presented as *n* (%)

*LA* left atrium, *LAA* left atrial appendage, *LV* left ventricle, *LVEF* left ventricular ejection fraction, *RoPE* risk of paradoxical embolism, *PFO* patent foramen ovale, *OAC* oral anticoagulants

### Patients with a major CSE

Characteristics of 11 patients who had a major CSE are presented in Table S6 (Electronic Supplementary Material). A major CSE was detected significantly more often in patients with major ECG abnormalities (*p* < 0.001): 10 (90.9%) of the 11 patients with a major CSE (all patients with LV aneurysm or LV thrombus) had major ECG abnormalities that required echocardiography regardless of stroke analysis. Such ECG abnormalities were present in 119 (11.1%) patients of the total study population. Only 1 (0.1%) patient without major ECG abnormalities had a major CSE (left atrial myxoma).

Out of 333 patients who had TTE during the 1st week after admission, 4 (1.2%) had a CSE. The risk of finding a major CSE during the 1st week was not significantly different from that of detecting a major CSE at a later point in time (*p* = 0.704), neither was the risk of finding an LV thrombus (*p* = 0.177).

## Discussion

In this study, TTE detected a major CSE in only 1% of 1084 patients with ischaemic stroke or TIA of undetermined cause. Notably, 10 of 11 patients with a major CSE also had major ECG abnormalities that warranted TTE assessment anyway. Our findings suggest that the current strategy of routine echocardiography in patients with ischaemic stroke or TIA of undetermined cause might be replaced by selective echocardiography in patients with major ECG abnormalities only. Additionally, contrast echocardiography should be performed in patients eligible for PFO closure [15]. With this strategy, the number of TTEs would be reduced by 73%.

In accordance with Dutch guidelines [16] we considered PFO screening appropriate in patients < 60 years old, with a RoPE score of ≥ 6 points. A PFO was detected in 23% of those patients who underwent contrast-enhanced echocardiography, similar to the estimated prevalence in healthy individuals but lower than the 40–50% reported in patients with ischaemic stroke of undetermined cause [17]. This discrepancy might reflect dissimilar patient populations and differences in prior diagnostic analyses.

The prevalence of TTE-detected major CSE in this study is lower than detection rates reported by most previous studies (0.3–37%) [18–26]. Several aspects may play a role. Most previous studies were retrospective, and patients were often selectively included after referral for echocardiography. Also, some studies included patients with known CSE. Another factor that can explain differences is a change of insights into treatment implications. For instance, it has been shown that a change to anticoagulant therapy does not lower recurrent stroke risk in the presence of poor LV function or complex aortic atheromas [10–12]. Furthermore, although sometimes reported as CSE, for mitral valve prolapse, mitral annular calcification and spontaneous echo contrast no causal relationship with stroke has been demonstrated [3, 4]. Notably, a previous retrospective study by our group [25] found a proportion of major CSE that would have been similar to our present rate, if we then had classified LV dysfunction as minor CSE.

Previous studies showed that cardiac symptoms and ECG abnormalities were the strongest predictors for detecting CSE [23, 25]. Our results corroborate these findings: major ECG abnormalities were present in the vast majority of patients with a major CSE. Although having major ECG abnormalities was not a pre-defined outcome measure, we consider this a highly relevant finding that deserves more attention in future studies. However, in order to assess the effect of TTE screening on clinical outcomes in a randomised controlled trial, a very high number of participants is needed to show a significant difference in clinical endpoints.

Studies of cardiac CT in ischaemic stroke patients reported higher detection rates of 3–17% [27–29]. However, these populations included patients with known CSE [27, 28]. Timing was also different: cardiac CT was often performed in the acute stroke setting, whereas TTE was performed at a later time. The study in patients with ESUS was retrospective and included 48% of eligible patients [29]. Moreover, several CSE other than thrombi are better detected with TTE because of its dynamic nature. In conclusion, (selective) cardiac CT might be of additional value in patients with ischaemic stroke or TIA of undetermined cause, but this approach needs confirmation by prospective data.

### Strengths and limitations

This is the first large-scale, prospective multicentre study to examine the yield of TTE in a well-defined population. Major CSE were defined as pathologies for which a change of therapy reduces the risk of recurrent stroke.

This study has several limitations. First, not all patients with ischaemic stroke or TIA of undetermined cause underwent TTE; for most patients a reason was stated. Furthermore, not all patients had MRI-proven ischaemic stroke; yet, the inclusion of patients with clinically suspected ischaemic stroke and TIA ensures that results are applicable to everyday practice. In addition, not every patient had intracranial vasculature imaging. Also, TOE was not performed routinely. The superiority of TOE compared to TTE in ischaemic stroke patients lies mainly in detecting left atrial (LA) and left atrial appendage (LAA) thrombi, complex aortic atheromas and valve vegetations [4]. Yet, LA and LAA thrombi are extremely rare in patients in sinus rhythm [30], complex aortic atheromas do not change therapy [11, 12], and a stroke in a patient with (suspicion of) endocarditis is no stroke of ‘undetermined cause’.

As the most recent guidelines on PFO treatment were published after enrolment had started [15], contrast echocardiography was not always performed when it currently would be indicated. In addition, specific patient characteristics and preferences might have prevented intravenous contrast injections. Finally, the median period until TTE assessment was 30 days, reflecting logistic capabilities of the participating hospitals. Although the effect of timing on TTE results is unknown, we cannot exclude the possibility that a thrombus may have dissolved at the time of echocardiography. Nonetheless, we observed no significant difference in major CSE detection with TTE during the 1st week as compared to a later point in time.

## Conclusions

In this large-scale multicentre observational study, routine TTE in patients with ischaemic stroke or TIA of undetermined cause revealed clinically relevant findings in 1% of all patients, the majority of whom also had major ECG abnormalities. We therefore suggest that the current routine referral strategy for echocardiography should be replaced by a selective strategy.

### Supplementary Information

Supplemental tables
