# Supplementary material for: Routine transthoracic echocardiography in ischaemic stroke or transient ischaemic attack of undetermined cause: a prospective multicentre study
Source: Neth Heart J. 2023 Oct 23;32(2):91–8. doi: 10.1007/s12471-023-01819-7 (PMC10834921; doi:10.1007/s12471-023-01819-7)
Supplement: Supplementary file 1 — Supplemental tables [file 12471_2023_1819_MOESM1_ESM.docx]

**Table S1** Affiliations

| **Hospital name** | **Patients (*n*)** | **Inclusion period** |
| --- | --- | --- |
| Academic University Medical Center, location AMC, Amsterdam | 54 | 01-01-2020 – 30-09-2020 |
| Albert Schweitzer Hospital, Dordrecht | 167 | 09-02-2019 – 30-09-2020 |
| Elisabeth-TweeSteden Hospital, Tilburg | 79 | 05-04-2019 – 30-09-2020 |
| Isala Hospital, Zwolle | 278 | 02-01-2019 – 30-09-2020 |
| Maasstad Hospital, Rotterdam | 31 | 06-02-2019 – 30-09-2020 |
| Medisch Spectrum Twente, Enschede | 475 | 05-03-2018 – 30-09-2020 |

**Table S2** Overview of cardiac sources of embolism and their corresponding implications for treatment

| Major cardiac sources of embolism | Treatment change |
| --- | --- |
| Endocarditis | Antibiotics, surgery in selected cases |
| Intracardiac tumour | Surgery |
| LV aneurysm | OAC |
| (Rheumatic) mitral valve stenosis | OAC |
| Thrombus in the LA, LAA, LV, or ascending aorta | OAC |
| Minor cardiac sources of embolism |  |
| Complex aortic arch atheromatous plaques |  |
| Dilated cardiomyopathy with LVEF <35% |  |
| Moderate or severe aortic valve stenosis | Valve surgery in selected cases* |
| Right-to-left shunt |  |
| Patent foramen ovale | Closure or OAC in selected cases |

LA = left atrium, LAA = left atrial appendage, LV = left ventricle, LVEF = left ventricle ejection fraction,

OAC = oral anticoagulants.

* Not preventive of recurrent stroke

**Table S3 Inter-rater reliability**

| **Category** | **κ** | **95% CI** | ***p*-value** |
| --- | --- | --- | --- |
| Major CSE | 0.885 | 0.725 - 1.045 | <0.001 |
| Minor CSE | 1.000 | 0.840 - 1.160 | <0.001 |

The Fleiss’ kappa test was used to estimate the inter-rater reliability (kappa (κ)) on the presence of a major CSE and minor CSE on 50 TTE’s, as reported by the original reviewer and two blinded, independent reviewers.

CI = confidence interval

**Table S4** Basic TTE characteristics

| **Variable** | **Total (*n* = 1084)** | **CSE (*n* = 11)** | **No CSE (*n* = 1073)** |
| --- | --- | --- | --- |
| LV dysfunction | 124/1083 (11.4%) | 8/10 (80.0%) | 116/1073 (10.8%) |
| Mild (LVEF 45-54%) | 90/1083 (8.3%) | 5/10 (50.0%) | 85/1073 (7.9%) |
| Moderate (LVEF 30-44%) | 25/1083 (2.3%) | 1/10 (10.0%) | 24/1073 (2.2%) |
| Severe (LVEF <30%) | 10/1083 (0.9%) | 2/10 (20.0%) | 8/1073 (0.7%) |
| LV wall motion abnormalities | 123/1073 (11.3%) | 9/11 (81.8%) | 114/1062 (10.7%) |
| LV diastolic dysfunction | 417/817 (51.0%) | 5/10 (50.0%) | 412/807 (51.5%) |
| Grade I | 352/817 (43.1%) | 4/10 (40.0%) | 348/807 (43.1%) |
| Grade II | 60/817 (7.3%) | 1/10 (10.0%) | 59/807 (7.3%) |
| Grade III | 5/817 (0.6%) | 0/10 (0.0%) | 5/807 (0.6%) |
| LA volume |  |  |  |
| Normal (<35 ml/m^2^) | 732/908 (80.6%) | 4/6 (66.7%) | 728/902 (80.7%) |
| Mildly abnormal (35-42 ml/m^2^) | 108/908 (11.9%) | 1/6 (16.7%) | 107/902 (11.9%) |
| Moderately abnormal (42-48 ml/m^2^) | 36/908 (4.0%) | 0/6 (0.0%) | 36/902 (4.0%) |
| Severe abnormal (>48 ml/m^2^) | 32/908 (3.5%) | 1/6 (16.7%) | 31/902 (3.4%) |
| Aortic valve calcification |  |  |  |
| None or mild | 1052/1060 (99.2%) | 8/8 (100.0%) | 1044/1052 (99.2%) |
| Moderate | 5/1060 (0.5%) | 0/8 (0.0%) | 5/1052 (0.5%) |
| Severe | 3/1060 (0.3%) | 0/8 (0.0%) | 3/1052 (0.3%) |

CSE = cardiac source of embolism, LA = left atrial, LV = left ventricular

**Table S5** Additional cardiac imaging in included patients

| Imaging modality | Number of patients |
| --- | --- |
| TTE | 1084 (100%) |
| TTE with agitated saline | 123 (11.3%) |
| TOE | 71 (6.5%) |
| Cardiac CT | 15 (1.4%) |
| Cardiac MRI | 27 (2.5%) |

TTE = transthoracic echocardiography, TOE = transoesophageal echocardiography, CT = computed tomography, MRI = magnetic resonance imaging.

**Table S6** Characteristics of patients with major cardiac sources of embolism

| **CSE** | **Sex/**  **age** | **Medical history** | **Clinical stroke characteristics** | **Brain imaging** | **ECG** |
| --- | --- | --- | --- | --- | --- |
| LV aneurysm | F  52 | TIA, myocardial infarction | Cortical infarction (R), rtPA and EVT  NIHSS 18 | CT: hyperdense vessel sign  CTa: proximal occlusion of right MCA | Previous infarction |
| LV aneurysm | F  68 | Myocardial infarction, | Cerebellar infarction  NIHSS 0 | CT: no recent ischaemia | Previous infarction |
| LV aneurysm | F  85 | Myocardial infarction (1993) | Cortical infarction (R), rtPA  NIHSS 10 | CTa: possible occlusion of right MCA | Previous infarction |
| LV aneurysm | M  71 | Myocardial infarction,  OHCA, ICD | Cortical infarction (L), EVT  NIHSS 4 | CTa: proximal occlusion of left MCA | Previous infarction |
| LV aneurysm | M  80 | Myocardial infarction (2x) | Cortical infarction (L), EVT  NIHSS 26 | CT: hyperdense left MCA CTp: ischaemia left MCA area  MRI: recent ischaemia left MCA area | Previous infarction |
| LV thrombus | M  52 | None | Cortical infarction (L), rtPA  NIHSS 1 | CTp: ischaemia left MCA area  CTa: distal occlusion of left MCA | Previous infarction |
| LV thrombus | M  65 | None | Cortical infarction (L), rtPA  NIHSS 5 | CTa: distal occlusion of left MCA | Previous infarction |
| LV thrombus | M  66 | CABG, ischaemic stroke (2x) | Cortical infarction (L), rtPA and EVT  NIHSS 23 | CTa: distal occlusion of left MCA | New LBBB |
| LV thrombus +  LV aneurysm | M  68 | CABG | Cortical infarction (R)  NIHSS 2 | CT: no recent ischaemia | Previous infarction |
| LV thrombus +  LV aneurysm | M  73 | Myocardial infarction (2x) | Two cortical infarctions (L)  NIHSS 1 | CT: recent ischaemia left frontal lobe, older ischaemia left parietal lobe | Previous infarction |
| Myxoma | F  75 | None | Subcortical TIA (L)  NIHSS 0 | CT: no recent ischaemia | Normal |

CABG = coronary artery bypass grafting, CSE = cardiac source of embolism, CT = computed tomography, CTa = CT angiography, CTp = CT perfusion, ECG = electrocardiogram, EVT = endovascular thrombectomy, ICD = implantable cardioverter defibrillator, LV = left ventricle, MCA = middle cerebral artery, MRI = magnetic resonance imaging, NIHSS = national institute of health stroke scale, OHCA = out of hospital cardiac arrest, rtPA = recombinant tissue plasminogen activator.
